# Supporting Mental Health During the COVID-19 Pandemic Using a Digital Behavior Change Intervention: An Open-Label, Single-Arm, Pre-Post Intervention Study

**DOI:** 10.2196/31273

**Published:** 2021-10-06

**Authors:** Charlotte Summers, Philip Wu, Alisdair J G Taylor

**Affiliations:** 1 DDM Health Coventry United Kingdom; 2 School of Management Royal Holloway, University of London Egham United Kingdom

**Keywords:** stress, mental health, COVID-19, digital therapy, mHealth, support, behavior, intervention, online intervention, outcome, wellbeing, sleep, activity, nutrition

## Abstract

**Background:**

The COVID-19 pandemic is taking a toll on people’s mental health, particularly as people are advised to adhere to social distancing, self-isolation measures, and government-imposed national lockdowns. Digital health technologies have an important role to play in keeping people connected and in supporting their mental health and well-being. Even before the COVID-19 pandemic, mental health and social services were already strained.

**Objective:**

Our objective was to evaluate the 12-week outcomes of the digitally delivered Gro Health intervention, a holistic digital behavior change app designed for self-management of mental well-being, sleep, activity, and nutrition.

**Methods:**

The study used a quasi-experimental research design consisting of an open-label, single-arm, pre-post intervention engagement using a convenience sample. Adults who had joined the Gro Health app (intervention) and had a complete baseline dataset (ie, 7-item Generalized Anxiety Disorder scale, Perceived Stress Scale, and 9-item Patient Health Questionnaire) were followed up at 12 weeks (n=273), including 33 (12.1%) app users who reported a positive COVID-19 diagnosis during the study period. User engagement with the Gro Health platform was tracked by measuring total minutes of app engagement. Paired *t* tests were used to compare pre-post intervention scores. Linear regression analysis was performed to assess the relationship between minutes of active engagement with the Gro Health app and changes in scores across the different mental health measures.

**Results:**

Of the 347 study participants, 273 (78.67%) completed both the baseline and follow-up surveys. Changes in scores for anxiety, perceived stress, and depression were predicted by app engagement, with the strongest effect observed for changes in perceived stress score (*F*_1,271_=251.397; *R*^2^=0.479; *P*<.001).

**Conclusions:**

A digital behavior change platform that provides remote mental well-being support can be effective in managing depression, anxiety, and perceived stress during times of crisis such as the current COVID-19 pandemic. The outcomes of this study may also support the implementation of remote digital health apps supporting behavior change and providing support for low levels of mental health within the community.

## Introduction

The COVID-19 pandemic has created an unprecedented global health challenge and has strained health care systems worldwide [1]. To minimize the risk of infection and spread, and to protect the most vulnerable groups of people and the population at large, governments across the world have advised people at high risk to leave their homes for very limited purposes [2].

There is risk that the COVID-19 outbreak will create a *second pandemic* of mental health crises across health systems and communities [3]. Prior to the COVID-19 pandemic, mental health and social services were already stretched. Depression is the second leading cause of disability worldwide, and by 2030, it is expected to be the leading contributor to the global burden of disease [4]. Efforts to contain the spread of COVID-19, including prolonged social distancing and self-isolation, may trigger or exacerbate social, mental, and physical health problems, such as anxiety, relationship breakdowns, domestic violence, substance abuse or withdrawal, and obesity [5-7]. This could be especially serious for those with preexisting medical and psychological conditions [8].

Times are unprecedented, and the COVID-19 pandemic has created global uncertainty. During times of uncertainty, people are more likely to be stressed, depressed, and anxious [9-12]. In 2014, when an outbreak of the Middle East respiratory syndrome coronavirus (MERS-CoV)‎ was reported, anxiety levels were associated with an increased perception of susceptibility to infection and social avoidance behaviors related to travel and being in public places [13]. Data originating from Wuhan city in the Hubei province of China, collected through the National Health Commission of China, showed that there was a correlation between the rapidly increasing numbers of confirmed cases and deaths and psychological problems, including anxiety, depression, and stress experienced by medical staff and the public [14].

The unpredictability and uncertainty of the COVID-19 pandemic and the resulting economic breakdown could increase the risk of mental health problems and exacerbate health inequalities [15]. Preliminary findings suggest adverse mental health effects in previously healthy people and in people with pre-existing mental health disorders [16]. Health care disparities will disproportionately affect socially disadvantaged patients, including those from ethnic minorities who have a relatively worse access to health care and receive poorer quality care than their Caucasian counterparts [17].

Approximately 5% of the general population experiences generalized anxiety disorder (GAD) at least once in their lifetime, and the estimated lag time to treatment for GAD can range between 9 and 23 years [18,19]. A key challenge to delivering interventions is low level of engagement [20,21].

Similarly, depression is the single largest contributor to global disability, with an estimated 300 million people affected worldwide [22]. Major depression has been found to impair the quality of life of people [23], as well as their psychosocial functioning [24]. However, resources to address these challenges are limited—for instance, the World Health Organization Mental Health Atlas 2017 reported a global median of 9 mental health workers per 100,000 people [25].

Face-to-face therapy and guided self-help techniques such as cognitive behavioral therapy and mindfulness have been shown to be effective in treating depression and anxiety [26-28]. However, face-to-face therapy traditionally provides point-in-time support, and there is struggle to scale fast enough to address growing mental health challenges [29]. With restrictions in interactions and activities during the COVID-19 pandemic, innovative delivery methods are required to augment care.

Our study aims to add to the research and evidence base on the effectiveness and engagement levels of a digital behavior change app (Gro Health) in the context of the COVID-19 pandemic. Previous research has shown that when mental health apps are properly designed, they can be cost-effective and scalable solutions for the treatment of anxiety, stress, and depression [30]. Moreover, meta-analyses of randomized controlled trials have shown that mental health apps can help alleviate symptoms of anxiety and depression, as well as assist patients to self-manage their conditions [31-34]. However, there is still limited research on the effectiveness of mental health apps in promoting behavior changes and improving health outcomes [35]. Health apps aiming at *lifestyle interventions* hold great promise, but evidence of their use and efficacy amid the COVID-19 pandemic is sparse [36].

To address this gap, we designed a quasi-experimental study to evaluate the 12-week outcomes of the Gro Health platform, a behavior change intervention for self-management of mental well-being, sleep, activity, and nutrition. The intervention provides education with modules such as stress management; building mental well-being and resilience; benefits of meditation; and guided activities to maintain positive mental well-being, including guided mindfulness-based meditations, classical music, 360-degree immersive guided relaxation videos, and facilitated yoga classes.

The primary study objective was to determine the effectiveness of delivering mental well-being activities using the Gro Health app on self-reported symptoms of anxiety (7-item Generalized Anxiety Disorder scale [GAD-7]), depression (9-item Patient Health Questionnaire [PHQ-9]), and perceived stress (Perceived Stress Scale [PSS]) [37-39]. We posited that greater engagement in the guided activities to maintain positive mental well-being (eg, yoga Tai Chi, Qi Gong, and guided mindfulness) would lead to improvements in anxiety, depression, and perceived stress compared to the baseline.

## Methods

### Intervention

Gro Health is a digital health intervention that provides behavioral change support through structured education and guided activities in the areas of mental well-being, nutrition, sleep, and activity. The app utilizes a similar behavior change architecture as the Low Carb Program, which has been demonstrated to achieve long-term engagement and sustainable behavior change [21].

Gro Health provides educational and therapeutic behavioral change support in the following four therapeutic areas: (1) mental well-being, (2) sleep, (3) activity, and (4) nutrition. Of particular interest for this study, engagement with the well-being/function of the app was examined. The well-being function of the app provides structured educational modules on topics such as stress management, building mental well-being and resilience, and benefits of meditation, delivered via video and text. These modules are supported with behavior change tools and resources, such as guided mindfulness-based meditations, Qi Gong, Tai Chi, classical music, 360-degree immersive guided relaxation videos, and facilitated yoga classes. Guided activities are of varying lengths (approximately 7-24 minutes long) and are presented in video and podcast format. Please see Multimedia Appendix 1 for example screenshots.

On March 1, 2020, the Gro Health app was updated to include education around minimizing the risk of infection and spread of COVID-19, in response to the feedback received from app users. The education syllabus is detailed in Table 1. The digital platform also provides digital tools for self-monitoring data activity (eg, steps and distance), body weight, blood pressure, heart rate, mood, food intake, body weight, and blood glucose levels. Participants can converse with coaches should they have questions and speak to peers in a moderated peer-to-peer community. Weekly automated feedback is provided to users based on their use of the program through email notifications, and participants are notified weekly to engage within the app. Prior studies demonstrate that peer support may help prevent stress and burnout, anxiety, and symptoms of depression [40-43]. The platform uses artificial intelligence to facilitate conversation and social support within the peer-to-peer support community by presenting community discussions to users that match their interests and demographics, including age, gender, and their self-selected goal.

**Table 1:** Syllabus of the COVID-19 educational program within the Gro Health app.

Module number	Title	Learning objectives
1	Safety notes	To ensure appropriate clinical safety context is provided and understood
2	Introduction to COVID-19	To understand what COVID-19 is, its origins, and current understandings
3	Symptoms of COVID-19	To define the symptoms of COVID-19 with the latest available evidence.
4	How to stay safe and prevent contracting and spreading the virus	To understand the protocol around social distancing
5	Washing your hands	To ensure washing hands is efficient and being completed often with the most effective technique
6	What to do if you feel unwell	To understand the latest protocol for illness during the COVID-19 pandemic
7	If I get infected with coronavirus, will I get better?	To share the latest available information about the COVID-19 pandemic, in particular the recovery rates
8	What to do if you need to go to hospital or see a doctor	To understand the latest protocol for hospital or doctor appointments during the COVID-19 pandemic
9	Living in self-isolation	Creating a routine to support social distancing while self-isolating (eg, food delivery services)
10	Managing stress	Understanding steps that can be taken to minimize stress levels during the COVID-19 pandemic
11	Mindfulness	Utilizing mindfulness-based practices to support well-being
12	Mental well-being	Understanding steps that can be taken to support mental and emotional well-being during the COVID-19 pandemic
13	What is meditation?	Understanding what meditation is and how it could be incorporated into a daily routine

The content and strategies used in the program are reviewed by primary care physicians and built off prior research and theory [21]. The program encourages participants to select a goal on registration (eg, lose weight, improve fitness, healthier life for family, reduce stress, improve dietary choices, be happier, and improve a health condition). Participants are periodically prompted to consider how close they are to attaining their goal.

### Study Design

The study used a quasi-experimental research design consisting of an open-label, single-arm, pre-post intervention. Ethics approval was obtained from the Royal Holloway, University of London ethics review board. Participants were not paid for their participation and accessed the Gro Health app for free. Participants downloaded the app and agreed to terms of service and privacy policy of the Gro Health app, which included consent to use anonymized data for research purposes. Minimal de-identified user data required for the analyses were collected.

### Study Participants

We collected a convenience sample of 347 participants aged 22-70 years (mean 49.6, SD 9.24 years) who signed up on the Gro Health app between February 26, 2020, and March 27, 2020. Just over half of participants were female (162/273, 59.3%). All participants were based in the United Kingdom. A total of 40.3% (110/273) of them had full-time employment, 68.1% (186/273) were White, and 80.2% (219/273) reported being obese. An a priori power analysis using G*Power (version 3.1; Heinrich-Heine-Universität Düsseldorf) indicated that a sample size of 270 people would be sufficient to detect a medium effect size (*r*=0.3) with 80% power, using a linear bivariate regression with α=.05. Thus, our proposed sample size of N=347 was more than adequate for detecting an effect of the linear predictor (well-being engagement) separately based on the outcomes of PHQ, GAD-7, and PSS score changes.

**Figure 1:**
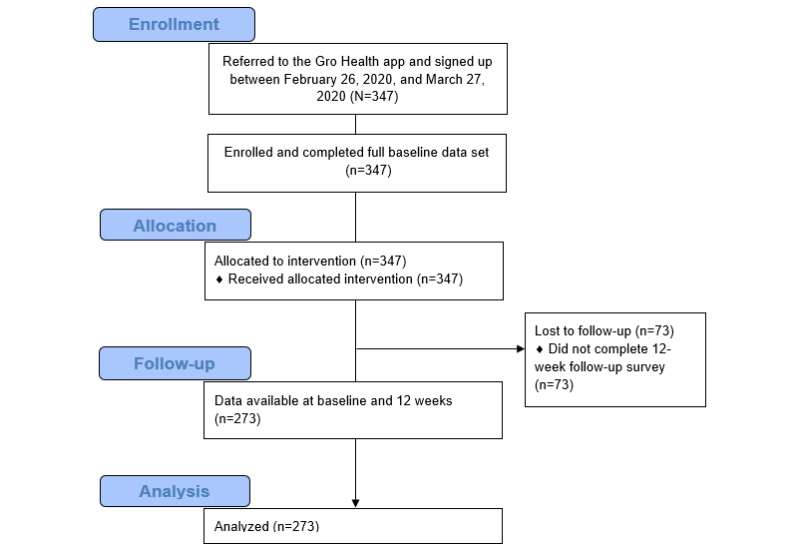
Participant flow chart used for this study.

See Table 2 for baseline characteristics and Figure 1 for the participant flowchart of the study. At the baseline, participants’ mean age was 49.6 (SD 9.2) years.

**Table 2:** Baseline characteristics of study participants (N=347).

Characteristic		Value
Age (years), mean (SD)		49.6 (9.2)
**Gender, n (%)**		
	Male	111 (40.7)
	Female	162 (59.3)
**Employment, n (%)**		
	Full-time employment	110 (40.3)
	Part-time employment	65 (23.8)
	Retired	82 (30)
	Student	1 (0.4)
	Unemployed	15 (5.5)

### Study Measures

Upon sign-up (baseline), participants (ie, app users) were asked to report their age, gender, health goal, and diagnosis of any pre-existing health conditions. They were also asked to complete the following scales: GAD-7, PHQ-9, and PSS. At 12 weeks, participants were asked to complete the same scales again, in the same format. User engagement with the Gro Health app was monitored and recorded as the total number of minutes of active engagement with the app across the 12-week study period. Of the 347 participants that provided full baseline data, 273 (78.67%) completed the follow-up surveys at 12 weeks.

### Statistical Analyses

Analyses were performed using the SPSS software (version 22.0; SPSS Inc). First, paired-sample *t*-tests were performed to compare mean changes in the three outcome measures (ie, anxiety, depression, and perceived stress), as measured by GAD-7, PHQ-9, and PSS, respectively, between the baseline and the 12-week follow-up. Second, a linear regression analysis was used to calculate how in-app engagement in the well-being function of the app (recorded in minutes) predicts participants’ change in mental health status, as well as changes in anxiety, depression, and perceived stress scores. Change scores for anxiety were calculated by subtracting follow-up anxiety scores from baseline anxiety scores, with a positive calculated score indicating a reduction in anxiety. Change scores for depression and stress were calculated in the same way. To control for potential effects of demographics and other health-related variables, age, gender, and COVID-19 self-diagnosis were included in the regression as control variables. Occupation status and ethnicity, included as multicategorical variables, were used as factors in one-way analysis of variance (ANOVA) with the three outcome measures. Bonferroni posthoc tests followed up any significant effects. Relevant statistical assumptions were assessed prior to the analysis. The normal distribution of the outcome measures was met, indicating the data was suitable for parametric analyses. Additionally, the assumptions of independence and normal distribution of residuals, linearity, and homoscedasticity were tenable, meaning the data were appropriate for a linear regression analysis.

## Results

### Overview

Of the 273 study participants who completed both the baseline and follow up surveys, 12.1% (n=33) reported that they had received a positive diagnosis of COVID-19. App engagement was measured through total minutes of use, an analytic indicator used in prior studies to evaluate the effective engagement of digital health apps [44,45]. The mean number of engaged minutes with the well-being function of the Gro Health app was 36.74 (SD 25.9) minutes, as recorded during the 12-week study period.

### Changes in Depression

Across the 12-week study period, there was a statistically significant change in PHQ-9 scores (reduction in score: mean 2.33, SD 2.97), which is a 32.95% reduction from the baseline mean score of 7.07 (SD 4.62) to the follow-up mean score of 4.74 (SD 3.82) (*t*_272_=15.6; 95% CI 2.04, 2.63; *P*<.001).

**Figure 2:**
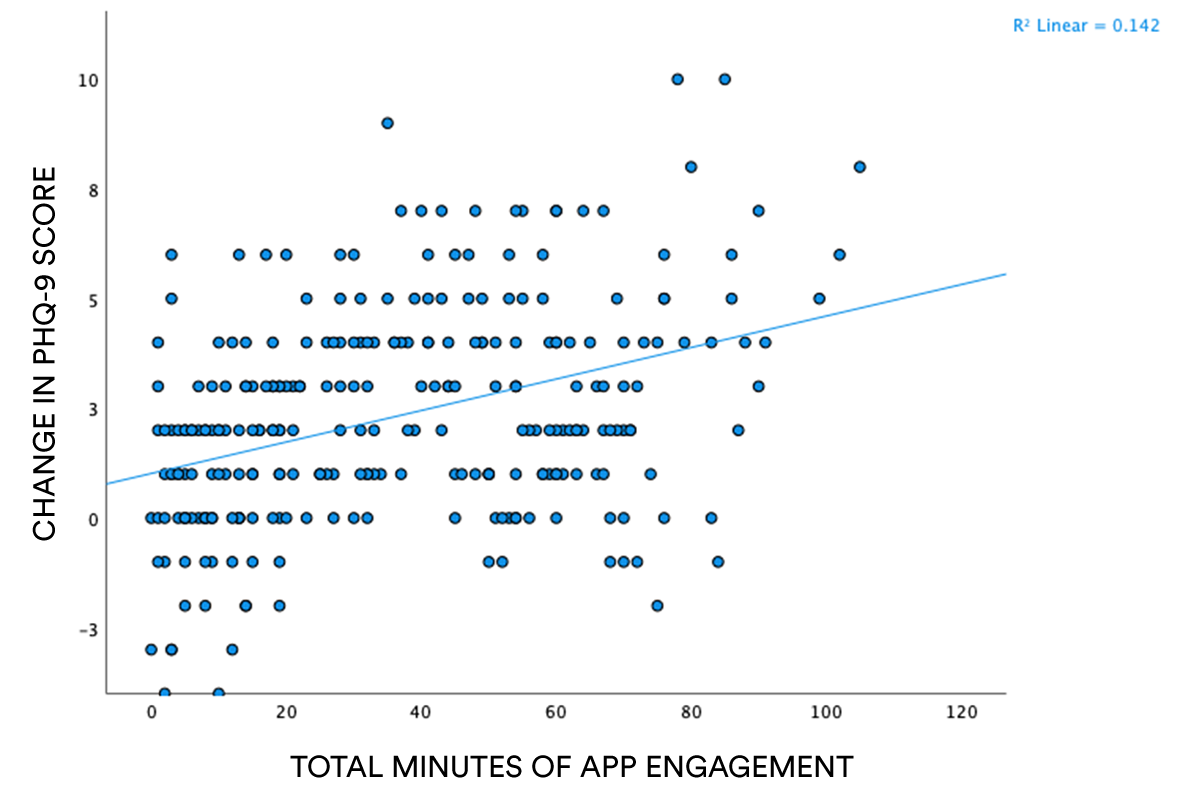
Scatter plot showing correlation between total time of engagement with the well-being function of the Gro Health app and pre-post test difference in PHQ-9 scores. PHQ-9: 9-item Patient Health Questionnaire.

As shown in Figure 2, a positive relationship exists between well-being engagement and change in PHQ-9 scores. This observation suggests that individuals who engaged for more time with the app also experienced the greatest reduction in markers of depression. A simple linear regression was calculated to predict participants change in PHQ-9 scores based on well-being engagement, while controlling for demographic and health-related variables. Demographic variables (eg, age and gender) and health-related variables (eg, COVID-19 diagnosis) were first entered into the model as controls, with Gro Health app engagement entered into a separate block as the predictor variable. The final model significantly accounted for 19.5% of the variance in PHQ-9 change scores (*R*=0.442; *R*^2^=0.195; *F*_1,271_=10.74; *P*<.001). Well-being engagement significantly positively predicted PHQ-9 change scores (β=.378; *t*_271_=6.8; 95% CI 0.026-0.046; *P*<.001), and it accounted for 14% of variance in PHQ-9 change scores when controlling for the demographic and prior COVID-19 diagnosis. For every additional minute of engagement with the app, PHQ-9 change scores increased by B=0.036 (unstandardized beta coefficient), implying that participants’ depression levels decreased when using the app.

A second linear regression was conducted to evaluate whether well-being engagement could predict PHQ-9 change scores in participants with higher levels of depression (n=50), that is, among app users with scores ≥12 [38]. The regression indicated that similar to the previous analysis, well-being engagement significantly positively predicted PHQ-9 change scores (β=.488; *t*_48_=3.654; 95% CI 0.021-0.073; *P*<.001) and that it accounted for 21% of variance in PHQ-9 change scores, when controlling for the demographic and prior COVID-19 diagnosis. For every additional minute of well-being engagement with the app, PHQ-9 change scores changed by B=0.047, meaning participants with higher engagement levels within the app saw a greater improvement in their PHQ-9 scores.

### Changes in Anxiety

Across the 12-week study period, there was a statistically significant change in GAD-7 scores (reduction: mean 2.18, SD 2.26), which is a 31.82% reduction from the mean baseline score of 6.85 (SD 3.25) to the mean follow-up score of 4.67 (SD 3.08) (*t*_272_=15.9; 95% CI 1.91-2.45; *P*<.001).

**Figure 3:**
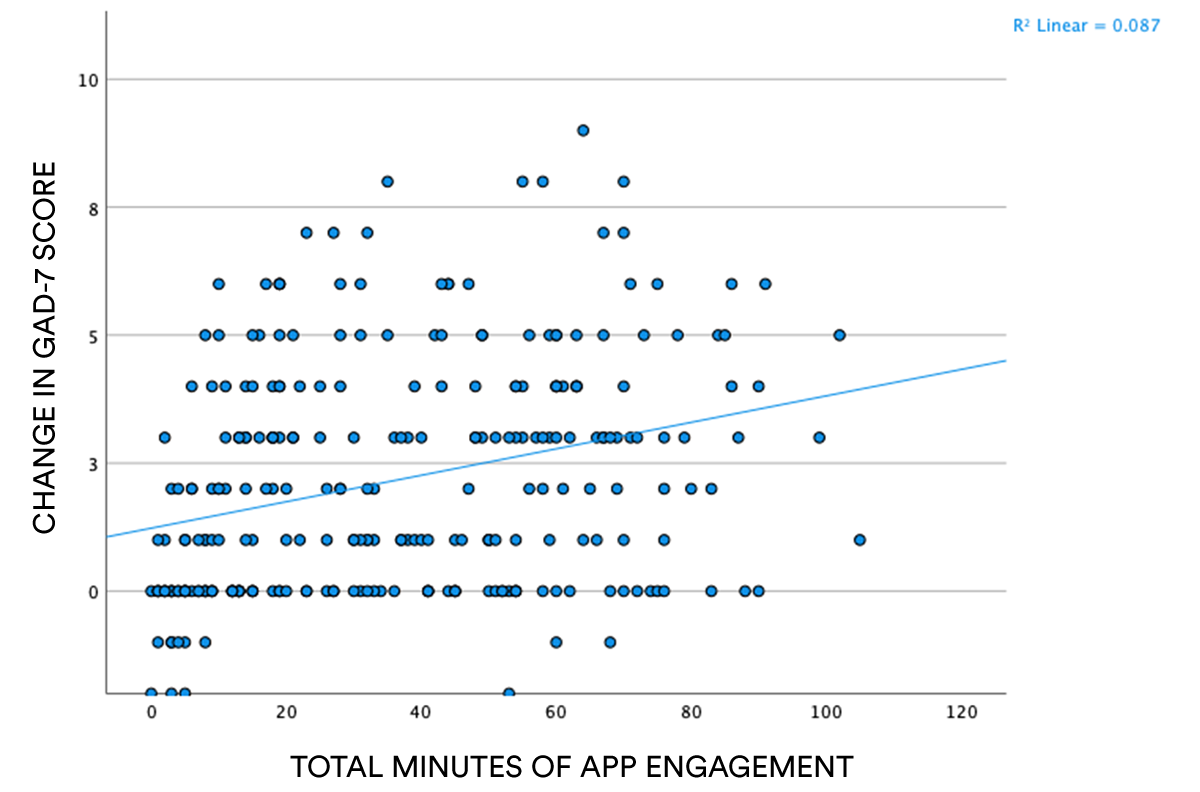
Scatter plot showing correlation between total time of engagement with the well-being function of the Gro Health app and pre-post test difference in GAD-7 scores. GAD-7: 7-item Generalized Anxiety Disorder scale.

As can be seen in Figure 3, a positive relationship exists between total minutes of app engagement and change in GAD-7 scores, suggesting that, as expected, those individuals who engaged more with the app were also those who experienced a greater reduction in anxiety levels.

A simple linear regression was performed to predict participants change in GAD-7 scores based on Gro Health app engagement, while controlling for demographic and health-related variables. As before, demographic and health-related variables were entered into the model first, followed by the Gro Health app engagement data. The final model significantly accounted for 11.9% of the variance in GAD-7 change scores (*R*=0.345; *R*^2^=0.11.9; *F*_6,272_=5.977; *P*<.001). Gro Health app engagement significantly positively predicted GAD-7 change scores (β=.287; *t*_271_=4.938; 95% CI 0.015-0.035; *P*<.001), and it accounted for 8% of variance in GAD-7 change scores, while controlling for the demographic and prior COVID-19 diagnosis. For every additional minute of engagement with the app, GAD-7 change scores increased by 0.025, implying that participants’ anxiety levels decreased when using the app. COVID-19 self-diagnosis (β=.137, *t*_271_=–2.3; 95% CI 0.138-1.772; *P*<.001) predicted change in GAD-7 scores. Individuals self-diagnosed with COVID-19 had greater reduction in anxiety scores (measured by GAD-7: B=0.955) after app engagement than those who were not self-diagnosed with COVID-19.

### Changes in Perceived Stress

Across the 12-week study period, there was a statistically significant change in perceived stress scores (mean 4.13, SD 3.03), which was a 23.95% reduction from the mean baseline score of 17.24 (SD 3.43) to the mean follow-up score of 13.11 (SD 2.87) (*t*_272_=22.4; 95% CI 3.77-4.5; *P*<.001).

**Figure 4:**
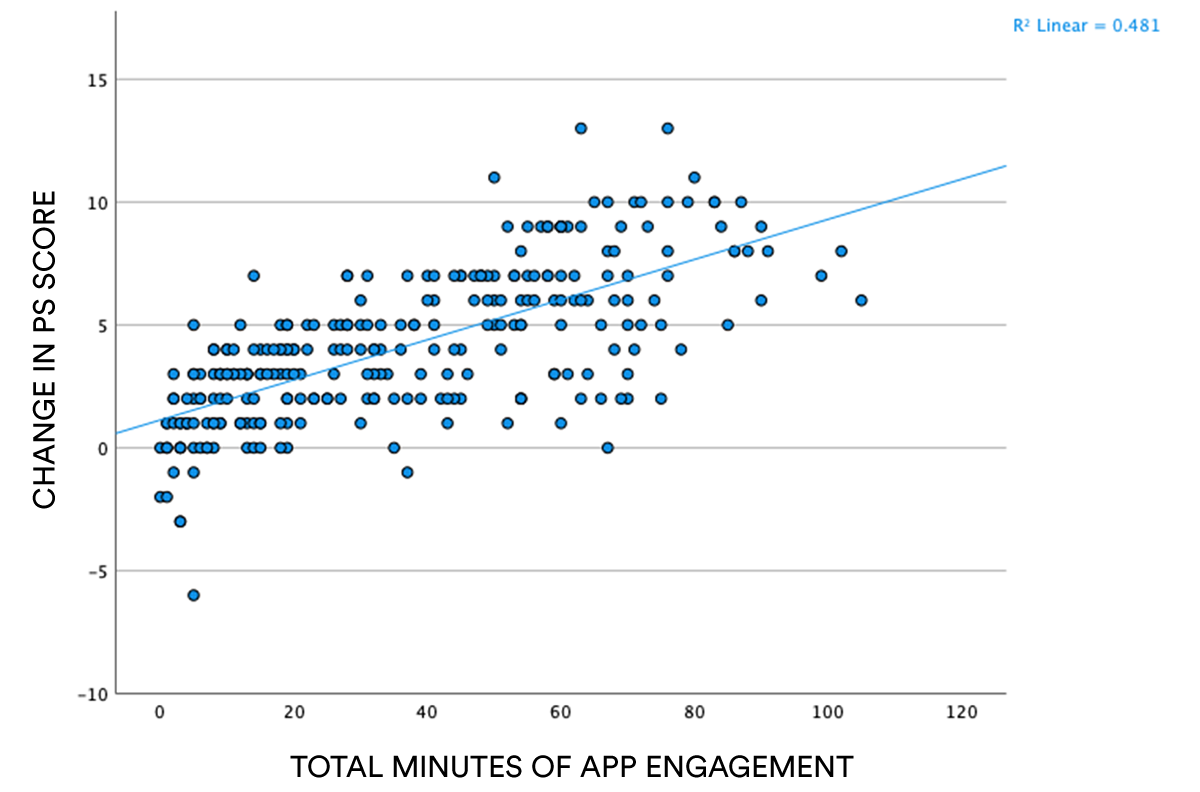
Scatter plot showing correlation between total time of engagement with the well-being function of the Gro Health app and pre-post test difference in PS scores. PS: perceived stress.

As described in Figure 4, a positive relationship exists between the total minutes of app engagement and change in PS scores, suggesting that, as predicted, those individuals who engaged more with the Gro Health app were also those who experienced a greater reduction in their stress levels.

A simple linear regression was performed to predict participants’ change in perceived stress scores based on Gro Health app engagement, while controlling for demographic and health-related variables. Similar to the previous analyses, demographics and prior COVID-19 diagnoses were entered into the model first, followed by the Gro Health app engagement predictor. The final model significantly accounted for 49% of the variance in perceived stress change scores (*R*=.70; *R*^2^=.49; *F*_6,272_=42.61; *P*<.001). Gro Health app engagement significantly positively predicted perceived stress change scores (β=.684; *t*_271_=15.461; 95% CI 0.07-0.091; *P*<.001), and it accounted for 45.8% of variance in PS change scores, while controlling for the demographic and prior COVID-19 diagnosis. For every additional minute of engagement with the app, perceived stress change scores increased by 0.081, implying that participants’ stress levels decreased when using the app.

A series of one-way ANOVAs were conducted to explore the potential effect of employment status on changes in PHQ-9, GAD-7, and PSS scores. The only significant effect found was for changes in perceived stress scores (*F*_2,270_=4.969; *P*<.01). A Bonferroni posthoc comparison revealed that change in perceived stress scores were significantly greater for retired participants (mean 5, SD 3.07) than for part-time and full-time individuals (mean 3.78, SD 3.05; *P*<.01). Therefore, those who were retired had greater improvements in perceived stress than presently employed. A series of one-way ANOVAs were conducted to explore potential differences in PHQ-9, GAD-7, and PSS changes scores as a function of ethnicity. The only significant effect found was for changes in PHQ-9 scores (*F*_3,269_=12.5; *P*<.001). A Bonferroni posthoc test found that White participants reported a significantly smaller change in PHQ-9 scores (mean 1.76, SD 2.25) than did participants of mixed ethnicity (mean 4, SD 1.52; *P*<.001) and those of Indian and Asian ethnicities (mean 3.45, SD 2.88; *P*<.001). Moreover, participants of mixed ethnicity had the greatest improvement in depression levels overall. Overall, Gro Health app engagement had the greatest impact on reducing stress scores (ie, change in perceived stress scores).

## Discussion

### Principal Findings

The results of this study are consistent with prior research on the use of digital health interventions for mental health, showing improvements in the symptoms of perceived stress, depression, and anxiety [46-48]. Over three-quarters (273/347, 78.67%) of the participants who signed up for the Gro Health app completed both the baseline and follow-up surveys. There were statistically significant interactions between app engagement and change in mental health scores, with the greatest effect observed in perceived stress scores.

The findings indicate that digital health solutions such as the Gro Health app that provide mental well-being resources could be of significant benefit when provided at scale to help address the growing mental health crises faced by global health services in the wake of the COVID-19 pandemic [49,50].

Greater changes in perceived stress scores were reported among participants who were retired compared to the rest of the population. The results from this study contradict prior evidence on the differences in employment status during the COVID-19 pandemic on mental health and well-being. Prior research evaluating employment and mental health during the initial stages of the pandemic found that compared to their unemployed counterparts, individuals who were employed at the start of the pandemic reported lower levels of mental health distress, higher levels of psychosocial well-being, better overall quality of life, and lower levels of overall loneliness, social loneliness, and emotional loneliness [51]. Further research should explore the variances in impact of this intervention in different employment groups.

There were differences in the impact of the app on depression scores between ethnic groups. For instance, individuals of Indian or Asian ethnicity reported greater improvements in their depression scores. This may be due to several factors, including the fact that mindfulness-based approaches may be more acceptable due to their grounding benefits in Eastern traditions [52]. Additionally, these results support prior research demonstrating that to engage people of various cultures and ethnicities within digital health solutions, they must be adapted to satisfy individual needs [53].

Individuals self-diagnosed with COVID-19 had greater reduction in anxiety scores after engaging with the app compared to those who were not self-diagnosed. Further research should explore whether the behavior change techniques provided by the app may help the longer-term mental health crisis that may occur in the aftermath of the pandemic both among those with a prior diagnosis of COVID-19 and those living with “long COVID.”

### Limitations

Our study has several limitations. We encouraged participants to engage in guided activities; however, we did not control for participants using other functionality of the app alongside the mental well-being tools. We also measured health outcomes (ie, PHQ-9, GAD-7, and PSS) by using patient self-report, rather than measuring those through medical records. However, previous research has found that these self-reported health outcomes can be quite close to actual values [54]. Similarly, patterns of engaged minutes with the app were not analyzed. Although outside the scope of this study, further analysis should examine whether patterns of app engagement impact the levels of change in mental health scores.

This was not a randomized controlled trial, so we cannot compare the 12-week results to a control or standard-of-care group. Therefore, the results of our trial should be interpreted cautiously because the study used convenience sampling, a single-arm design, and pre-post self-reported outcomes. Criticism for convenience sample use includes the lack of control over potential intervening variables (such as other stress-mitigating or mental well-being–boosting activities) that active participants may have been engaging in. Since the sample is not representative of the population, these results are not representative of the entire population. The study results have low external validity. However, a significant proportion of participants were from hard-to-reach populations (eg, retired, unemployed, and ethnic groups) or those diagnosed with type 2 diabetes, hypertension, or high cholesterol. Individuals with these health conditions are more at-risk of COVID-19 and its complications, which makes this sample of particular interest [55].

The sample contained 33 participants who self-reported a COVID-19 diagnosis. As the research context is set in the pandemic, analysis of covariance (ANCOVA) was conducted to evaluate whether app use had a greater effect on those who had COVID-19. Mixed results were obtained from these analyses. When app engagement time was controlled, a statistically significant difference was observed with regard to GAD-7 scores (*P*=.004) between the two groups, but not with regard to PHQ-9 and PSS scores.

Despite several limitations, the study suggests a digital platform that provides remote mental well-being support can be extremely effective in managing depression, anxiety, and perceived stress levels during times of crisis such as the current COVID-19 pandemic. Future studies with 6- and 12-month post-tests will provide a stronger assessment of the impact of the intervention on mental health outcomes.

Further research should explore the most appropriate mechanisms by which such digital health interventions can be scaled to help manage and mitigate the mental health demands that will inevitably follow a global pandemic of the magnitude of COVID-19 [49,50]. Additional research should also evaluate the use of the intervention in patients diagnosed with long-COVID. Emerging evidence suggests 20% of patients diagnosed with COVID experience symptoms of long COVID, including anxiety, breathlessness, and fatigue [56]. Furthermore, studies should explore whether the behavior change techniques recommended by the Gro Health app could also help alleviate the symptoms or burden of long COVID.

Although our design does not support causal conclusions, further research should investigate the particular in-app guided activities that might have an impact on users’ perceived stress, depression, and anxiety scores. In addition, it is important to conduct further research into identifying for which participants self-guided digital interventions may be sufficient in supporting mental well-being, and those who may need triaging to receive additional face-to-face or intensive support.

### Conclusions

A digital platform providing remote mental well-being support (Gro Health) can be effective in managing depression, anxiety, and perceived stress during times of crisis, such as the current COVID-19 pandemic. Further research should investigate how best to implement such digital health solutions at scale to mitigate the burden on national health services during pandemics.

## Appendix

Multimedia Appendix 1 Gro Health app screenshots showing well-being function, educational modules, and mindfulness-based behavior change activity.

## Abbreviations

ANCOVA: analysis of covariance
ANOVA: analysis of variance
GAD: generalized anxiety disorder
GAD-7: 7-item Generalized Anxiety Disorder scale
MERS-CoV: Middle East respiratory syndrome coronavirus
PHQ-9: 9-item Patient Health Questionnaire
PSS: Perceived Stress Scale
