# Supplementary figures and images for: Supporting Mental Health During the COVID-19 Pandemic Using a Digital Behavior Change Intervention: An Open-Label, Single-Arm, Pre-Post Intervention Study
Source: JMIR Form Res. 2021 Oct 6;5(10):e31273. doi: 10.2196/31273 (PMC8496681; doi:10.2196/31273)

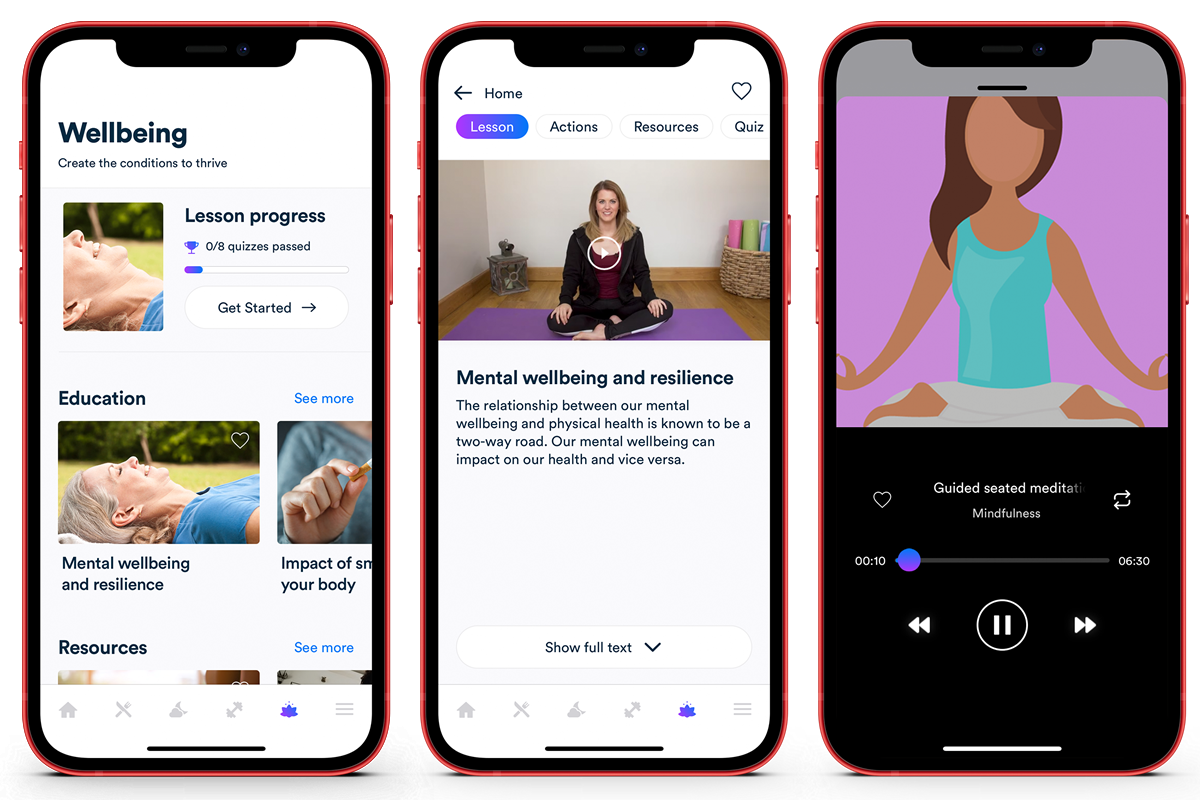

Supplement: Multimedia Appendix 1 [file formative_v5i10e31273_app1.png]
